# Cytotoxic Activity and Memory T Cell Subset Distribution of *in vitro*-Stimulated CD8^+^ T Cells Specific for HER2/neu Epitopes

**DOI:** 10.3389/fimmu.2019.01017

**Published:** 2019-05-09

**Authors:** Maria Kuznetsova, Julia Lopatnikova, Julia Shevchenko, Alexander Silkov, Amir Maksyutov, Sergey Sennikov

**Affiliations:** ^1^Laboratory of Molecular Immunology, State Budgetary Scientific Institution Research Institute of Fundamental and Clinical Immunology, Novosibirsk, Russia; ^2^State Research Center of Virology and Biotechnology VECTOR, Koltsovo, Novosibirsk, Russia; ^3^Novosibirsk State University, Novosibirsk, Russia

**Keywords:** CD8^+^ T cells, cytotoxicity, memory T cell subsets, HER2/neu, stem cell-like memory T cells

## Abstract

Minimal residual disease remaining after resection of primary tumors can lead to tumor recurrence and metastasis, increasing mortality and morbidity rates among cancer patients. Thus, there is a need for new technologies for recognition and elimination of single cancer cells remaining in a patient's body after radiation therapy, chemotherapy, or surgical resection. Effector CD8^+^ T cells, also commonly known as cytotoxic T lymphocytes (CTLs), play a key role in antitumor cellular immunity and, when properly activated, are able to effectively destroy tumor cells. The aims of this study were to obtain CD8^+^ CTLs specific for the HER2/neu epitopes E75 and E88 and to assess the cytotoxic activity and composition of these cells in terms of the distribution of memory T-cell subsets. We obtained HER2-specific CD8^+^ T cells and assessed T cell subset distribution among them including naive T cells (T_N_), central memory T cells (T_CM_), effector memory T cells (T_EM_), stem cell-like memory T cells (T_SCM_) and terminally-differentiated T cells (T_EMRA_) via eight-color flow cytometry. HER2-specific CTLs were largely (~40–50%) represented by T_SCM_ cells, a population capable of mounting pronounced antitumor immune responses due to a combination of effector function and self-maintenance. In comparison with activated peripheral blood mononuclear cells (PBMCs) and bulk CD8^+^ T cells, HER2-specific CTLs exhibited greater cytotoxicity against the HER2-expressing human breast adenocarcinoma cell line MCF-7 and produced higher levels of IFN-γ in response to tumor cells. We also showed the presence of HER2-specific CTLs in healthy individuals and increase in them in HER2-positive breast cancer patients. Collectively, our results suggest that HER2-specific CD8^+^ T cells isolated using this approach could be used for adoptive T-cell transfer to eliminate tumor cells and prevent metastasis and relapse in patients with HER2-overexpressing cancers.

## Introduction

Every arm of the immune system is involved in the antitumor immune response. Both CD8^+^ cytotoxic T-lymphocytes (CTLs) and CD4^+^ T-helper cells play important roles in anti-tumor immunity by secreting perforin and granzyme, inducing FasL/TRAIL-mediated cell death, or secreting effector cytokines such as interferon gamma (IFN-γ) and tumor necrosis factor alpha (TNF-α) (1–3). Both T-helper cell and CTL frequencies are associated with better survival in cancer patients (4, 5). However, CD8^+^ CTLs have received greater attention in the field of cancer Immunotherapy. When properly activated by antigen-presenting cells (APCs) displaying antigenic peptides in complex with major histocompatibility complex (MHC) class I molecules, CTLs are able to specifically recognize and destroy malignant cells (6). Therefore, CD8^+^ T-lymphocytes are used more often for adoptive T-cell transfer studies (5). It is clear that the ability to directly mediate cytotoxicity is one of the main reasons that CD8^+^ T-cells are preferred for adoptive T-cell therapy. However, cytotoxicity is not the only requirement for adoptively-transferred cells; the ability of transferred CTLs to maintain themselves is equally important for the induction of a stable response to T-cell therapy. The viability and proliferation of T cells directly correlate with the antitumor activity of adoptive T-cell therapy (7–9). In this regard, the presence of memory T-cells in the transferred T-cells is of particular importance (10).

The tumor-associated antigen HER2/neu is a member of the human epidermal growth factor receptor family and is expressed during embryogenesis as well as on normal tissue cells of adult organisms (11, 12). Overexpression of HER2/neu occurs in various malignant carcinomas, which has made it a convenient target for immunotherapy. Many studies and approaches have been designed to inhibit the proliferation of HER2-positive tumor cells, some of which have been applied in clinical practice (13–19). Despite the success of several of these approaches, expression of HER2/neu continues to be associated with aggressive cancers, poor clinical prognoses and deteriorating survival rates (20). Therefore, studies of HER2/neu therapeutics are clearly still relevant (12, 21–24).

We have previously shown that a CTL response against HER2-expressing MCF-7 cells can be induced in a culture of activated peripheral blood mononuclear cells (PBMCs) using dendritic cells (DCs) transfected with a polyepitopic DNA construct encoding antigenic HER2/neu peptides (19). However, the potency of the immune response generated was low. We hypothesized that an insufficient number of HER2/neu-specific effector cells were contained in the mixed culture of PBMCs. In an attempt to increase the potency of the antitumor immune response, we decided to purify enriched populations of activated HER2/neu-specific CTLs. In the present study, we obtained HER2-specific CTLs using transfected DCs and HLA Streptamer technology and investigated the effector properties of the resulting cells, as well as their composition in terms of the distribution of memory T-cell subsets.

## Materials and Methods

### DNA Constructs

A pMax-2 polyepitopic DNA construct encoding multiple copies of each of the two target HER2/neu epitopes in different molecular contexts was produced and designated as the “HER2 plasmid.” The two target immunogenic epitopes were HER2/neu p369–377 (E75, KIFGSLAFL) and HER2/neu p689–697 (E88, RLLQETELV).

A control plasmid DNA construct (pDNA5-BC-C) encoding the non-immunogenic peptide Ag51 was produced and designated as the “P5 plasmid.”

Both plasmids were designed and described in more detail previously (23).

### Donors and Patients

PBMCs were obtained from 18 HLA-A^*^02-positive healthy donors (age 34 to 60 years, median 42 years), as well as four HLA-A^*^02-positive patients with a confirmed diagnosis of HER2-positive breast cancer receiving treatment at Oncology Department No. 3, Novosibirsk City Clinical Hospital No. 1 (age 29 to 65 years, median 55 years). The presence of the HLA-A^*^02 allele was assessed by HLA genotyping, which was carried out at the stage of selecting donors and patients.

**Figure 1:**
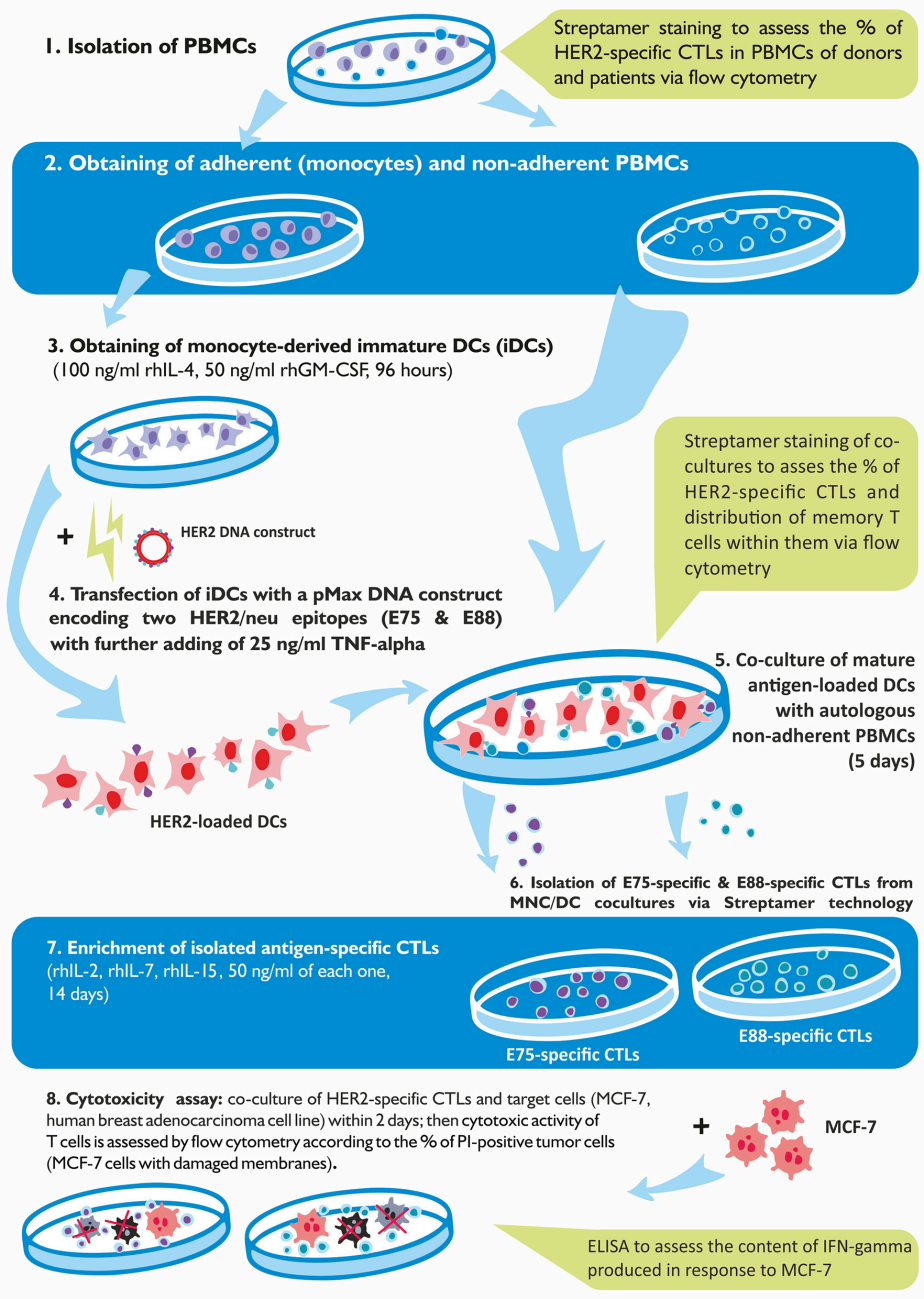
Schematic representation of the experimental design of the study.

Voluntary informed consent was obtained from all donors and patients. This study was approved by the local Research Institute of Fundamental and Clinical Immunology (RIFCI) ethics committee. The experimental design of the study is shown in Figure 1.

PBMCs were cultured in RPMI-1640 medium supplemented with 10% fetal calf serum, 2 mM L-glutamine, 50 μM mercaptoethanol, 25 mM HEPES, 80 μg/mL gentamicin and 100 μg/mL ampicillin (hereinafter called the culture medium). HER2-specific CTLs were obtained using a protocol we developed earlier (22) with a number of methodological changes described later. After magnetic separation, the epitope-specific CD8^+^ T cell population was cultured in the presence (50 ng/mL each) of IL-2, IL-7 and IL-15 (Biozol, Germany) for 2 weeks in order to enrich the purified cells and stimulate memory T-cell proliferation.

### Isolation of Adherent PBMCs

Adherent PBMCs were isolated by incubation for 30 min on plastic Petri dishes (Nunc, Denmark) containing 10 mL of culture medium at 37°C under a humidified atmosphere containing 5% CO_2_. After incubation, the medium containing non-adherent PBMCs was transferred into a clean centrifuge tube. Next, 10 mL of RPMI-1640 medium was added to the Petri dishes and adherent PBMCs were removed from the bottom of the Petri dishes with a scraper (Sigma-Aldrich, USA). Suspensions containing adherent and non-adherent fractions were centrifuged at 1,500 rpm for 10 min. After centrifugation, the cell pellets were resuspended in culture medium for further manipulations.

In order to identify the optimal conditions for purifying adherent PBMCs, we previously conducted an analysis of six variations of the method in which incubation time and bottom pretreatment were variables. These variations involved cultivation in untreated or bovine serum albumin (BSA)-pretreated Petri dishes for 30, 60, or 120 min. The effectiveness of each strategy was assessed by labeling adherent cells with phycoerythrin (PE)-conjugated anti-human CD14 antibody (Becton Dickinson, USA) and counting the relative number of CD14^+^ cells on a BD FACSVerse flow cytometer.

### Selection of Transfection Method

To determine the optimal method for plasmid delivery to DCs, we compared three transfection methods: (i) magnet-assisted transfection, (ii) electroporation and (iii) nucleofection of monocyte-derived DCs from healthy donors. A PMaxGFP plasmid encoding green fluorescent protein (GFP) was used for the comparison. Transfection efficiency was assessed based on the number of GFP-positive cells 24 h post-transfection using flow cytometry. DCs were analyzed by flow cytometry in the region of large granular leukocytes.

The nucleofection procedure was carried out using the Amaxa Human Dendritic Cell Nucleofector Kit (Lonza, Switzerland) according to the manufacturer's protocol and a Nucleofector 2b instrument. Magnet-assisted transfection of DCs was carried out using magnetic “MATra-A” nanoparticles and a universal 26 × 26 cm magnetic board for magnetic transfection (PromoKine, USA) according to the manufacturer's protocol. Electroporation was performed using Bio-Rad Gene Pulser disposable cuvettes (0.2 cm electrode gap) and a BTX ECM 830 electroporator with a voltage of 260 V and a pulse duration of 5 ms.

### Transfection of Immature DCs

Nucleofection was chosen for DC loading according to the results of the selection experiment. The HER2 plasmid was used for generation of HER2-loaded DCs (DC_HER2_). The P5 plasmid was used as a transfection control, yielding p5-loaded DCs (DC_p5_).

### Maturation of Transfected DCs and Activation of HER2-Specific T Cells

DC_HER2_ and DC_p5_ cell cultures (1 × 10^6^ cells/mL) were cultured in flasks with a surface area of 25 cm^2^ in culture medium containing 25 ng/mL TNF-α for 24 h to stimulate maturation of DCs. After 24 h of incubation with TNF-α, the cell number and viability of transfected cultures were counted in a Goryaev chamber using erythrosine staining. Next, the antigen-loaded DCs were co-cultured with autologous non-adherent PBMCs to generate HER2-specific T cells. Co-culture (PBMC+DC) was performed at a PBMC:DC ratio of 10:1 in 48-well plates (Cellstar, USA) containing culture medium (1 mL/well) for 4 days.

Co-cultures were labeled with antigen-specific Streptamers (IBA, Germany) to determine the frequencies of E75- and E88-specific CTLs. A detailed staining protocol has been previously described (23). Co-cultures were also analyzed for frequency of memory T-cell subsets as well as a number of effector functions.

### Phenotyping of HER2-Specific CTLs

Co-cultures of PBMCs and DCs stained with PE-conjugated Streptamers were labeled with the following monoclonal antibodies for flow cytometry: CD8-Brilliant-Violet-510, anti-human CD28-FITC, anti-human CD27-PerCP, anti-human CD95-PE-Cy7, anti-human CD127-APC, anti-human CD62L-APC-Cy7, anti-human CD45RA-Pacific-Blue. The prepared samples were analyzed on a BD FACSVerse. The lymphocyte gate was defined according to forward and side light scattering parameters. Then, CD8^+^ cells and Streptamer^+^CD8^+^ cells were gated from the lymphocyte gate events.

The following subsets were identified within the CD8^+^ and Streptamer^+^CD8^+^ lymphocyte populations: (i) T_EMRA_ (CD45RA^+^CD62L^−^); (ii) T_EM_ (CD45RA^−^CD62L^−^); (iii) T_CM_ (CD45RA^−^CD62L^+^); (iv) T_N_ (CD45RA^+^CD62L^+^CD127^+^CD27^+^CD28^+^CD95^−^); and T_SCM_ (CD45RA^+^CD62L^+^CD127^+^CD27^+^CD28^+^CD95^+^). Differentiation of T_EMRA_, T_EM_, and T_CM_ cell subsets was then analyzed via expression of CD27 and CD28.

### Analysis of HER2-Specific CTL Effector Properties

The target cells used to assess the direct cytotoxicity of HER2-specific T-lymphocytes were human breast adenocarcinoma MCF-7 cells (Bank of Cell Cultures, the Institute of Cytology of the Russian Academy of Medical Sciences, Russia). Evaluation of direct cytotoxicity was carried out according to a previously described protocol (23). Briefly, carboxyfluorescein succinimidyl ester (CFSE)-labeled MCF-7 cells were co-cultured with HER2-specific T-lymphocytes in wells of 96-well round-bottom culture plate at a ratio of 1:10 for 48 h and then labeled with propidium iodide (PI) and analyzed on a BD FACSVerse.

To quantitate cytotoxicity, we used the following formula:

$$\begin{array}{l} \%Cytotoxicity=\%PI^{+}CFSE^{+}MCF-7_{\left(effector+target\right)}-\\ \;\%PI^{+}CFSE^{+}\;MCF-7_{\left(target\right)}, \end{array}$$

where **% PI**^**+**^**CFSE**^**+**^**MCF-7**_(effector+target)_ was the percentage of non-viable MCF-7 cells among the total cells in a co-culture of effector cells and target cells and **% PI**^**+**^**CFSE**^**+**^ **MCF-7**_(target)_ was the percentage of target cells undergoing spontaneous death in the absence of effector cells.

IFN-γ production in response to target cells was evaluated in conditioned media using the commercial gamma-INTERFERON-IFA-BEST system (Vector-Best, Russia) according to the manufacturer's protocol. Conditioned media were harvested after 48 h of co-culture of E75- and E88-specific cells with MCF-7 cells.

### Statistical Analysis

Statistical analysis and graphical representations were done using Prism 6 (GraphPad Software). Statistical significance was determined using two-way ANOVA with Tukey's multiple comparisons test (when analyzed groups have passed D'Agostino & Pearson omnibus normality test) and nonparametric two-tailed Mann–Whitney test and Kruskal-Wallis or Friedman test with Dunn's multiple comparison test.

All the data are presented as median and interquartile interval.

## Results

### Optimization of the Adherent PBMC Purification Method

We compared six isolation methods for adherent PBMCs (30-, 60-, and 120-min incubation on untreated or BSA-treated plastic) by estimating the number of cells expressing the monocyte marker CD14. The results showed that incubation on untreated surfaces for 30 min yielded the highest numbers of CD14^+^ cells from PBMCs of healthy donors (57.12% of CD14^+^ cells, compared with 48.38 and 46.01% after 60- and 120-min incubation on untreated surfaces, respectively, and 53.30, 45.67, and 44.63% after 30-, 60-, and 120-min incubation on BSA-treated surface, respectively; *p* ≤ 0.05, *n* = 12, Kruskal-Wallis test with Dunn's multiple comparison test). Thus, to obtain adherent cells, we used a 30 min incubation on untreated plastic.

### DC Transfection

Comparison of DC transfection methods showed that the percentage of DCs expressing GFP following nucleofection significantly exceeded the percentage of GFP^+^ DCs following magnet-assisted transfection or electroporation (41.75% GFP^+^ DCs for nucleofection vs. 31.50% for magnet-assisted transfection and vs. 6.52% for electroporation, *p* = 0.0173 and *p* = 0.0022, respectively Mann-Whitney test). Thus, nucleofection was used for antigen loading of DCs. The viability of the DCs transfected using nucleofection with HER2/p5 plasmid did not decrease below 80% for both DC_HER2_ and DC_p5_, according to the cell viability assessment in a Goryaev chamber using erythrosine staining.

### The Frequency of E75- and E88-Specific CTLs Among PBMCs of Healthy Donors and Patients With HER2-Positive Breast Cancer

To confirm the *in vivo* formation of a specific immune response against HER2/neu, we examined the content of E75- and E88-specific CTLs among PBMCs from healthy donors and patients with HER2-overexpressing breast cancer.

**Figure 2:**
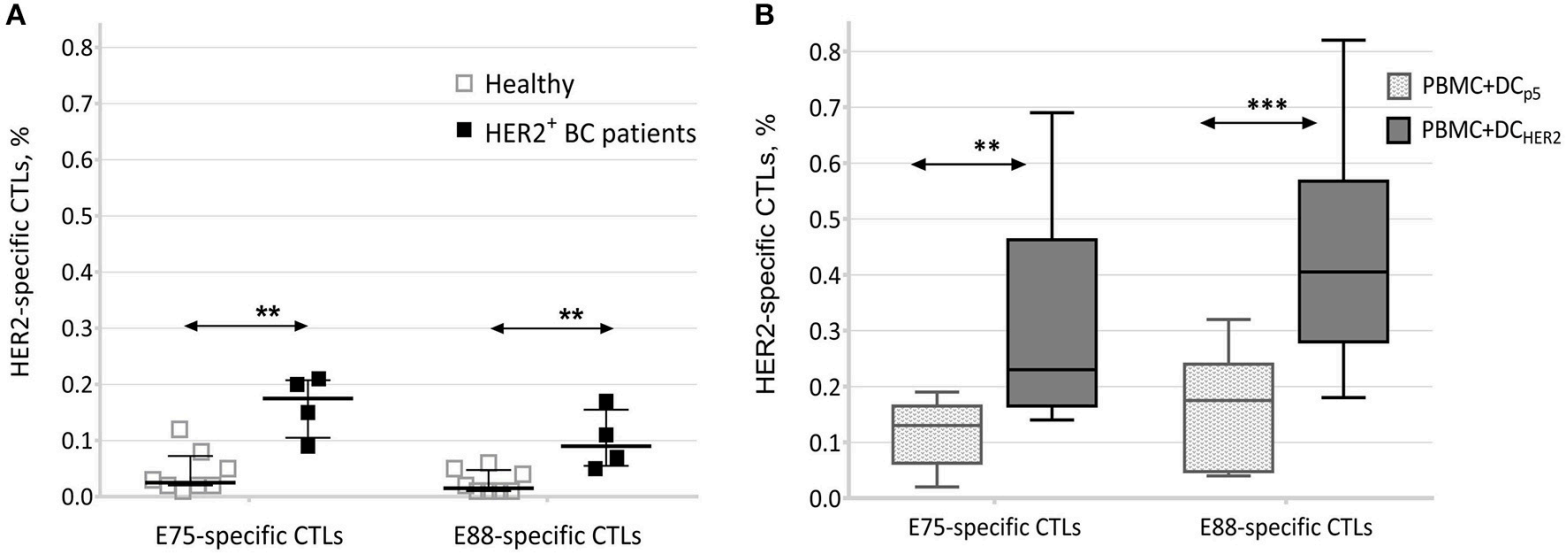
Frequencies of E75- and E88-specific CTLs. **(A)** Relative frequencies of HER2-specific CTLs among PBMCs from healthy donors (*n* = 8) and patients with HER2-positive breast cancer (*n* = 4). Data are presented as median and interquartile interval. The arrows indicate statistically significant differences, ^**^p ≤ 0.01 (Mann-Witney test). **(B)** Relative frequencies of E75- and E88-specific CTLs in co-cultures of PBMCs and DCs from healthy donors (*n* = 10). Data are presented as median, interquartile range, minimum and maximum. The arrows indicate statistically significant differences, ^**^p < 0.01, ^***^p < 0.001 (Repeated measures two-way ANOVA, Tukey's multiple comparison test). **PBMC+DC**_**p5**_-co-culture of PBMCs and DCs transfected with P5 plasmid; **PBMC+DC**_**HER2**_-co-culture of PBMCs and DCs transfected with the HER2 plasmid.

We found that PBMCs from HER2-positive breast cancer patients contained significantly higher proportions of HER2-specific CTLs (both E75-specific and E88-specific) compared with those of healthy donors (Figure 2A).

This result indicated that in association with development of HER2/neu-overexpressing tumors, clonal expansion of HER2-specific T-lymphocytes occurs, confirming the formation of a specific T-cell response to this antigen.

### Frequencies of E75- and E88-Specific CTLs in Co-cultures of PBMCs and Antigen-Loaded DCs

Analysis of co-cultures of PBMCs and DCs transfected with the HER2 plasmid (PBMC+DC_HER2_) showed that co-culture resulted in an increase in the frequencies of E75-specific T-lymphocytes (average, 0.32%; median, 0.23%) and E88-specific T-lymphocytes (average, 0.44%; median, 0.41%). These frequencies significantly exceeded those observed from co-cultures of PBMCs and DCs transfected with the P5 plasmid (PBMC+DC_p5_) (Figure 2B).

This result confirmed that clonal expansion of epitope-specific T lymphocytes was directly related to lymphocyte activation by DCs transfected with the HER2 plasmid, and that epitope-specific T cells could not be activated non-specifically by DCs transfected with a control plasmid.

### Distribution of T Cell Subsets Among DC-Stimulated CTLs

DC-stimulated E75 and E88-specific CTLs and total CD8^+^ T cell populations were analyzed by flow cytometry and classified according to phenotype into the following subpopulations: T_N_, T_SCM_, T_CM_, T_EM_, and T_EMRA_ (25–28).

**Figure 3:**
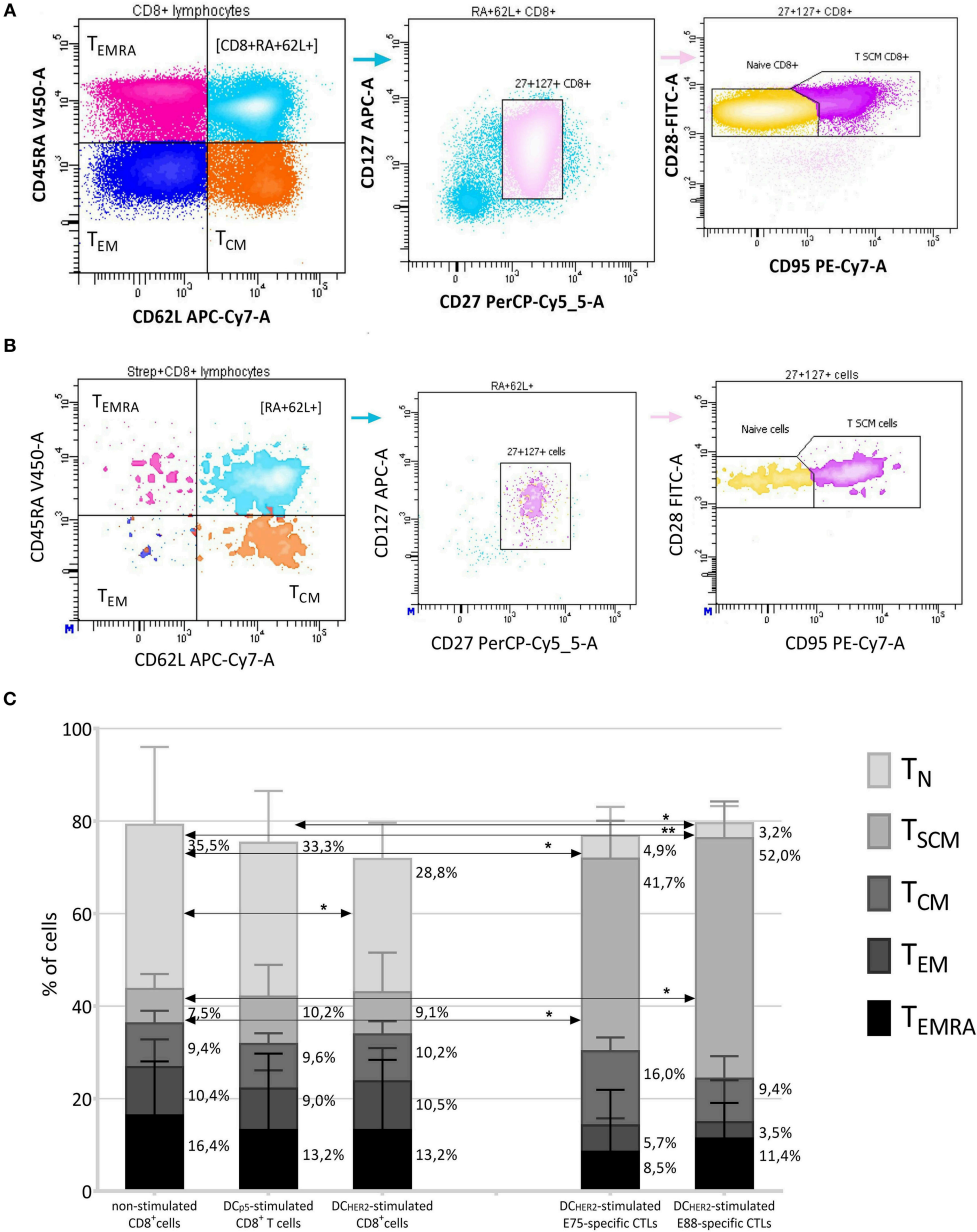
Phenotyping of HER2-specific CTLs. **(A,B)** The gating strategy for evaluation of the T-subset distribution within CD8^+^ T cells **(A)** and Streptamer^+^CD8^+^ T cells **(B)**. CD8^+^/Streptamer^+^CD8^+^ cells were divided into four populations according to the expression of CD45RA-V450 and CD62L-APC-Cy7, thus identifying subpopulations of T_EMRA_ (CD45RA^+^CD62L^−^), T_EM_ (CD45RA^−^CD62L^−^), T_CM_ (CD45RA^−^CD62L^+^), and CD45RA^+^CD62L^+^ cells. From the CD62L^+^CD45RA^+^ gate, CD127^+^CD27^+^ events were selected (thus identifying the CD45RA^+^CD62L^+^CD127^+^CD27^+^ population) and among them, the T_N_ cells (CD45RA^+^CD62L^+^CD127^+^CD27^+^CD28^+^CD95^−^) and T_SCM_ cells (CD45RA^+^CD62L^+^CD127^+^CD27^+^CD28^+^95^+^) were identified according to their staining by anti-CD95-PE-Cy-7 and anti-CD28-FITC antibodies. **(C)** Median frequencies of T subsets within total CD8^+^ T cells (three left columns) and within HER2-specific CTLs (two right columns). T subsets investigated: T_N_ (CD8^+^CD45RA^+^CD62L^+^CD127^+^CD27^+^CD28^+^CD95^−^), T_SCM_ (CD8^+^CD45RA^+^CD62L^+^CD127^+^CD27^+^CD28^+^CD95^+^), T_CM_ (CD8^+^CD45RA^−^CD62L^+^), T_EM_ (CD8^+^CD45RA^−^CD62L^−^), T_EMRA_ (CD8^+^CD45RA^+^CD62L^−^). The data are presented as median and interquartile interval (upper quartiles are showed). The arrows indicate statistically significant differences, ^*^p ≤ 0.05, ^**^p < 0.01 (Friedman test with Dunn's multiple comparison test, *n* = 6).

The typical contour plots showing the gating strategy for phenotyping the CD8^+^ T cells and CD8^+^ HER2-specific (Streptamer^+^) T lymphocytes, and the frequencies of the listed subsets are shown in Figure 3.

When comparing the frequencies of T-cell subsets among CD8^+^ T cells, no significant differences were observed between non-*in vitro* activated CD8^+^ T cells (Figure 3C, first column from left), DC_p5_-stimulated CD8^+^ T cells (Figure 3C, second column from left) and DC_HER2_-stimulated CD8^+^ T cells (Figure 3C, third column from left), except for T_N_ cells, which significantly decreased among total CD8^+^ T cells after co-culture with DC_HER2_ cells. Thus, a trend toward CD8^+^ T-lymphocyte activation was observed in DC_HER2_-activated CD8^+^ T-lymphocytes compared with non-activated cells, which was manifested in a reduction of T-_N_ cells.

Among E75- and E88-specific CD8^+^ T cells (two rightmost columns in Figure 3C), similar patterns were observed in the distribution of T-cell subsets (i.e., no significant differences between E75- and E88-specific CTLs), although both differed from bulk non-stimulated CD8^+^ T-cells. E75- and E88-specific CD8^+^ T cells contained significantly fewer T_N_ cells (4.9% among E75-specific CTLs, and 3.2% among E88-specific CTLs; 35.5% among non-stimulated CD8^+^ T cells, 33.3% among DC_p5_-stimulated CD8^+^ T cells and 28.8% within DC_HER2_-stimulated CD8^+^ T cells) and significantly more T_SCM_ cells (41.6% among E75-specific CTLs, and 52.0% among E88-specific CTLs; 7.5% among non-stimulated CD8^+^ T cells, 10.2% among DC_p5_-stimulated CD8^+^ T cells and 9.1% within DC_HER2_-stimulated CD8^+^ T cells).

**Figure 4:**
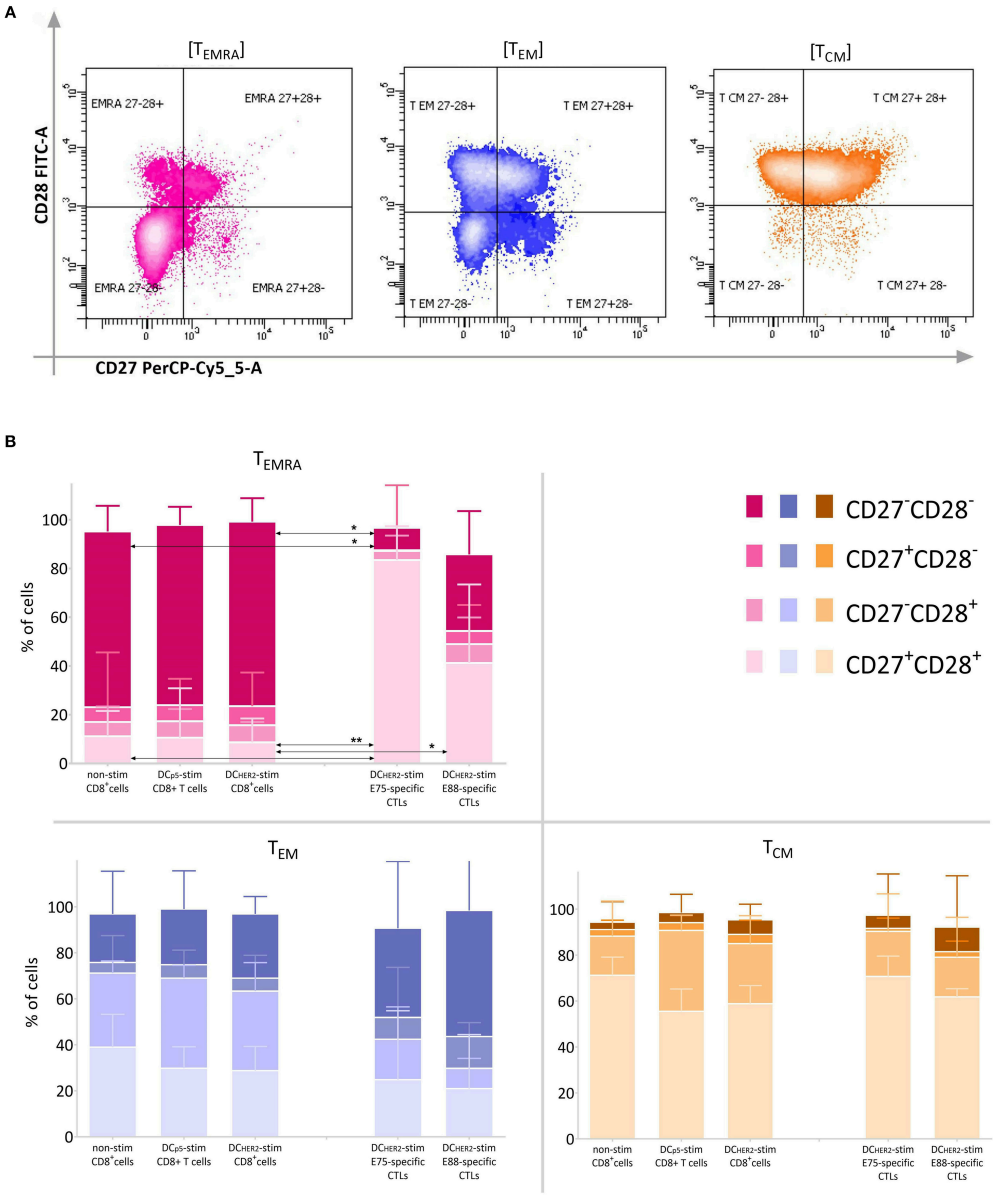
Expression of CD27 and CD28 within the T_EMRA_, T_EM_ and T_CM_ subsets. **(A)** Among the T_EMRA_ (CD45RA^+^CD62L^−^), T_EM_ (CD45RA^−^CD62L^−^), and T_CM_ (CD45RA^−^CD62L^+^) populations, expression of CD27 and CD28 was analyzed according the fluorescence in the CD27-PerCP-Cy5.5 and CD28-FITC channels. **(B)** Relative frequency of cells expressing CD27 and CD28 within T_EMRA_, T_EM_, and T_CM_ CD8^+^ T cell subsets (three left columns in each graph) and HER2-specific CTLs (two right columns in each graph).The arrows indicate statistically significant differences, without asterisks *p* < 0.1, ^*^p < 0.05, ^**^p < 0.01 (Friedman and Dunn's multiple comparisons test, *n* = 6).

A comparison of CD27 and CD28 expression within the T_EMRA_, T_EM_ and T_CM_ subsets is shown in Figure 4.

T_EMRA_ cells among the total CD8^+^ T cell population are mostly double-negative cells, reflecting a high degree of cell differentiation. However, T_EMRA_ cells among HER2-specific CTLs were characterized by a high frequency of CD27^+^CD28^+^ cells and a low frequency of CD27^−^CD28^−^ cells.

T_CM_ cells were mostly represented by CD27^+^CD28^+^ cells within all populations investigated. T_EM_ cells were represented in comparable parts by CD27^+^CD28^+^, CD27^−^CD28^+^, and CD27^−^CD28^−^ cells, and by a minor population of CD27^+^CD28^−^ cells; this was true for all three populations of CD8^+^ T cells. However, among HER2-specific CTLs there was a trend toward increased frequency of CD27^−^CD28^−^ cells compared with bulk CD8^+^ T cells.

In summary, the approach used to activate HER2-specific CTLs using DCs loaded with HER2 epitopes led to a decrease in the frequency of CD8^+^ T_N_ cells. The distribution of T-cell subsets within DC_HER2_-activated E75-specific CTLs was not significantly different from that of DC_HER2_-activated E88-specific CTLs. However, the distribution of subsets within both populations of HER2-specific T cells revealed a significant reduction of T_N_ cells and increase in T_SCM_ cells compared with bulk CD8^+^ T cells.

### Evaluation of the Effector Properties of HER2 Epitope-Specific CTLs

**Figure 5:**
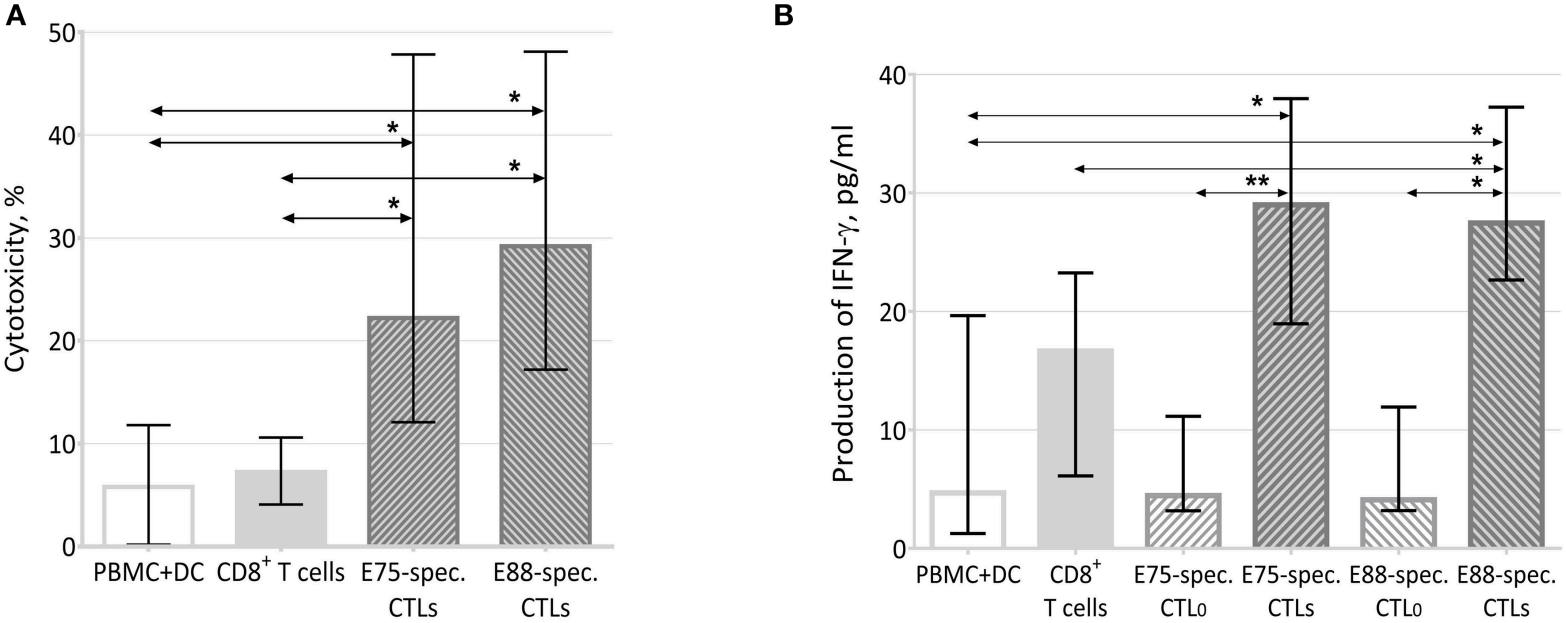
Effector properties of HER2 epitope-specific CTLs. **(A)** Cytotoxic activity of effector T-cells against the MCF-7 cell line (*n* = 12). **PBMC+DC** represented a co-culture of MCF-7 cells with a PBMC and DC co-culture; the latter were negative cells after sorting E75- and E88-specific CTLs from PBMC+DC_HER2_ co-cultures. **CD8**^**+**^ **T-cells** represented a co-culture of MCF-7 and CD8^+^ cells sorted from a co-culture of PBMCs and DCs. **E75-specific CTLs** represented a co-culture of MCF-7 cells with E75-specific CTLs sorted from PBMC+DC_HER2_ co-cultures. **E88-specific CTLs** represented a co-culture of MCF-7 cells with E75-specific CTLs sorted from PBMC+DC_HER2−_ co-cultures. The arrows indicate statistically significant differences, ^*^p < 0.05 (Kruskal-Wallis test with Dunn's multiple comparison test, *n* = 12). **(B)** IFN-γ in conditioned media from 48-h co-cultures of effector cells with MCF-7 target cells. **MNC+DC** refers to conditioned media from co-cultures of MCF-7 cells with PBMC+DC co-cultures; the latter were negative cells after sorting E75- and E88-specific CTLs from PBMC+DC_HER2_ co-cultures. The arrows indicate statistically significant differences, ^*^p < 0.05, ^**^p < 0.01 (Munn-Whitney test, *n* = 6). **CD8**^**+**^ **T cells** represented conditioned media from co-cultures of MCF-7 and CD8^+^ cells sorted from PBMC+DC co-cultures. **E75-specific CTL**_**0**_ represented conditioned media from cultures of E75-specific CTLs sorted from PBMC+DC_HER2_ co-cultures in the absence of MCF-7 cells. **E75-specific CTLs** represented conditioned media from co-cultures of MCF-7 and E75-specific CTLs. **E88-specific CTL**_**0**_ represented conditioned media from cultures of E88-specific CTLs sorted from PBMC+DC_HER2_ co-cultures in the absence of MCF-7 cells. **E88-specific CTLs** represented conditioned media from co-cultures of MCF-7 and E88-specific CTLs.

Analysis of the cytotoxic activity of E75- and E88-specific CTLs showed that the intrinsic cytotoxicity of the cells against HER2^+^ MCF7 cells was 29.20% for E88-specific cells and 22.20% for E75-specific CTLs. These values significantly exceeded the intrinsic cytotoxicity of control cultures of PBMCs and DCs (5.78%) and of CD8^+^ cells (7.25%) (Figure 5A).

Thus, HER2 epitope-specific T-lymphocytes had increased cytotoxicity against the MCF-7 cell line.

To evaluate one of the potential cytotoxicity mechanisms, we estimated the concentration of IFN-γ in conditioned media from 2-day effector cell cultures with and without the MCF-7 tumor cell line (see Figure 5B).

After 48 h of co-cultivation with target cells, the IFN-γ content in media from culture of E75- and E88-specific CTLs increased by more than 6-fold compared with CTL_0_ samples that did not contain target cells (from 4.46 to 29.00 pg/mL for E75-specific CTLs and from 4.10 to 27.47 pg/mL for E88-specific CTLs). The concentration of IFN-γ in the culture media of E75-specific CTLs and E88-specific CTLs significantly exceeded that of PBMC+DC co-cultures (6.36 pg/mL) as well as CD8^+^ T cells (16.66 pg/mL).

## Discussion

In this study, we selected immunogenic HER2 epitopes with the highest affinities for HLA-A02 alleles, which therefore are most effectively recognized by T-lymphocytes: E75 (HER2 p369–377, KIFGSLAFL) and E88 (HER2 p689–697, RLLQETELV) (13). We chose the HLA-A02 alleles since these are the most common alleles in the human population (29). Moreover, analysis of the distribution of HLA-A alleles among human populations on five continents showed that A^*^02:01 is one of the few HLA class I alleles found in a large proportion of human populations (30).

Among the breast cancer patients initially recruited according to HER2 positive expression status criterium (1+ /2+ /3+), there were both patients in remission and progression, as well as patients after complex treatment and surgery, and previously untreated patients. The final group of 4 patients included two patients after complex treatment and surgery and two patients with primary tumors. All of them were HER2 (3+). It also should be noted that in clinical practice the HER2 expression status is always analyzed before the HER2-targeted therapy.

Our evaluation of HER2-specific CTL frequency in the peripheral blood of healthy donors and patients with HER2-overexpressing breast cancer confirmed the formation of a specific immune responses against HER2/neu during the malignant process, despite the fact that this antigen is an epidermal growth factor receptor and is normally present on healthy epithelial cells of a number of organs (17). In 2004, Woll and co-authors showed that the frequency of CD8^+^ T cells specific for the HER2/neu E75 epitope was about 0.2–0.3% of bulk peripheral blood lymphocytes in patients with HER2-positive breast cancer (31). These data are consistent with our results. However, we were also interested in the question of whether the ~0.2% of HER2-specific CTLs in patient blood resulted from the development of a specific cellular response due to the HER2/neu overexpression, or, on the contrary, was similar to that of healthy individuals. Our comparison of patients and healthy donors made it possible to clarify that an increase in CTL clones specific to HER2/neu epitopes does occur in patients with breast cancer.

Correct antigen presentation plays a crucial role in immune response generation. The simplest approach for *in vitro* loading of APCs with antigen is the addition of an antigenic peptide to a cell culture, ensuring the capture and processing of the antigen by the MHC II pathway. The major advantage of this approach is the simplicity of the loading protocol. The use of nucleic acids as a source of antigen is also a common method of APC loading. If tumor material is sufficient, mRNA can be isolated directly from tumor cells and used for transfection of APCs. DNA, however, is more stable and is easier to work with it than with RNA (32–34). A number of studies using DCs transfected with non-viral DNA vectors have shown that loaded DCs can activate an effective antitumor immune response (7, 23, 33, 35). An advantage of nucleofection over other non-viral DNA delivery strategies is the fact that nucleofection allows delivery of molecules not only to the cytoplasm, as for other non-viral transfection methods, but also directly into the cell nucleus (36, 37).

In addition to the choice of the APC antigen loading strategy, there is also a choice of the cell type, hopefully with the most potent antitumor effects, which should be used for adoptive T-cell immunotherapy. Several studies have used one cell type as effector cells—either CD4^+^ T cells or CD8^+^ lymphocytes (16, 38, 39)—while others have used mixed cultures of T-helper and T-killer populations or bulk PBMCs (4, 40, 41). Many studies have emphasized that subpopulations of T cells play a synergistic role in orchestrating the immune response. In this regard, interest is growing in the role of T helper cells in the context of antitumor immunotherapy. Wolf and co-authors suggested the forthcoming appearance of cellular therapies that specifically combine CD8^+^ with CD4^+^ T cells and their testing in phase III clinical trials (5).

At the same time, adoptive transfer of pure populations of antigen-specific circulating CTLs or tumor-infiltrating T-cells has not lost its relevance (6, 16, 23). Despite the generation of an effective antitumor immune response, the cytotoxic properties of CTLs can be reduced due to the presence of other, non-effector cell types in the cell preparation (3). Our results also confirmed the increased effectiveness of specific antitumor immune responses when using pure populations of antigen-specific T-cells. Cultures of E75- and E88-specific cells are more than 90% composed of CD8^+^ T cells of a given antigen specificity (23). These populations have a more pronounced cytotoxic effect against HER2-expressing MCF-7 cells, produce more IFN-γ in response to target cells, and in addition, and have increased frequencies of memory cells compared to bulk CD8^+^ cells (both unstimulated *in vitro* and stimulated with DC_p5_ cells or DC_HER2_ cells).

The primary method for assessing peripheral blood T cell heterogeneity is flow cytometry. The main phenotypic characteristic of memory T cells is considered to be the appearance of the CD45RO^+^ isoform instead of the CD45RA^+^ isoform. In combination with these markers, the CD62L and CCR7 molecules, mediating homing into lymphoid organs, are used to identify memory subsets within CD8^+^ T cells (26, 27). It should be noted that there are currently no commonly accepted phenotypic criteria for dividing memory cells into subsets.

CD8^+^ T_CM_ cells, producing a large amount of IL-2 but low levels of effector cytokines, have the CD45RA^−^CD45RO^+^CCR7^+^CD62L^+^ phenotype. These cells differ from T_N_ cells only by the CD45 isoform, and are capable of homing to lymphoid organs. CD8^+^ T_EM_ cells, which produce high levels of IFN-γ and contain perforin and granzyme granules, differ from T_CM_ cells in their lack of expression of CCR7 and CD62L.

Adding two more important markers to the panel (the CD27 and CD28 molecules responsible for T-cell costimulation) allows assessment of T-cell differentiation level. As an example, some researchers consider cells with the phenotype CD45RO^+^CCR7^−^CD62L^−^CD27^−^CD28^−^ as effector memory cells and CD45RO^+^CCR7^−^CD62L^−^CD27^−^CD28^+^ cells as “transitional” memory T-cells; the latter are more differentiated than T_CM_ cells but less differentiated than T_EM_ cells according to a number of phenotypic characteristics (42, 43) and based on their proliferation in response to IL-15 *in vivo* (44, 45). Terminally-differentiated CD45RA^+^CCR7^−^CD62L^−^ T-cells negative for CD27 and CD28 are described as short-lived CTLs with pronounced effector properties that can be activated under the influence of non-professional APCs, but die immediately after executing their cytotoxic function (46).

T_SCM_ cells were first discovered in 2005 in the peripheral blood of mice in a study of experimental graft-vs.-host disease (47) and were later identified in human peripheral blood as well (48, 49). The principal difference in these cells from T_N_ cells was their high expression of CD95 and CD122; in common with T_N_ cells, they expressed CCR7, CD45RA, CD62L, CD27, CD28, and CD127 (IL-7Ra) but were completely negative for CD45R0 (49).

The panel of antibodies we selected for eight-color flow cytometry included antibodies against human CD8, CD45RA, CD62L, CD27, CD28, CD95, and CD127 as well as fluorochrome-conjugated antigen-specific reagents (Streptamers, Iba, Germany). This panel allowed us to identify all major circulating CD8^+^ T cell subsets among the E75- and E88-specific CTL populations.

A significant decrease in the frequency of T_N_ cells among E75- and E88-specific CTLs, as well as among bulk DC_HER2_-activated CD8^+^ cells, indicated that DCs transfected with the HER2 plasmid could effectively activate specific T-cell cytotoxic immune responses. It is also noteworthy that the largest component of total HER2-specific cells was T_SCM_ subset. This population is characterized by pronounced effector properties and long-term self-maintenance, which has attracted interest in these cells for cancer immunotherapy (27, 49, 50). T_SCM_ cells are known to be the least differentiated of all memory subsets, but at the same time are able to secrete IFN-γ, IL-2, and TNF-α in response to α-CD3/α-CD2/α-CD28 stimulation (49). These properties make T_SCM_ cells promising for further use in adoptive T-cell therapy. We attribute the increase in T_SCM_ frequency to the method for antigen-specific T-cell isolation we used: the isolated HER2-specific T-lymphocytes were cultured in the presence of IL-2, IL-7, and IL-15 (50 ng/mL each) (23). IL-2 is necessary to maintain the viability of T-lymphocytes, and IL-7 and IL-15 play a key role in triggering *in vitro* proliferation, in addition to signals from the T-cell receptor and costimulatory molecules. As mentioned earlier, T_SCM_ cells are characterized by high expression of CD122 (total β-chain of the receptor for IL-2 and IL-15) and CD127 (IL-7Rα).

We demonstrated a six-fold increase in IFN-γ production by DC_HER2_-activated E75- and E88-specific CTLs in response to target cells, which is consistent with a reduction in the number of T_N_ cells and an increase in T_SCM_ content within these populations. In addition, IFN-γ production by HER2-specific CTLs was almost 2-fold higher than that of activated bulk CD8^+^ cells of all other specificities. Thus, some level of IFN-γ production occurs in antigen non-specific T-cell immune responses, but IFN-γ production is significantly higher in antigen-specific T cells that executing their cytotoxic function.

## Conclusion

In this study, we demonstrated the presence of HER2-specific T-lymphocytes in the blood of healthy donors and a significant increase in their frequency in the blood of patients with HER2-overexpressing breast cancer. We showed the effectiveness of a technique to isolate CTL populations specific for HER2/neu epitopes. The resulting E75- and E88-specific CTLs consisted of more than 40% of T_SCM_ cells. These cells exhibited pronounced cytotoxicity and a higher level of IFN-γ production against the HER2-expressing tumor line MCF-7 cells compared with activated PBMCs. We expect that this approach will be effective for obtaining antigen-specific T-cells for adoptive T-cell transfer. Specifically, we assume that the cells obtained in this way can be effective for the elimination of HER2-expressing tumor cells in patients with HER2-overexpressing tumors after resection of the primary tumor, which may help in preventing of relapses and metastasis.

## Ethics Statement

This study was carried out in accordance with the Declaration of Helsinki with written informed consent from all subjects. The protocol was approved by the local ethics committee of Research Institute of Fundamental and Clinical Immunology (RIFCI).

## Author Contributions

MK contributed to the design, experimental work, and optimization of each experimental stage, analysis, and interpretation of data, drafting of the manuscript, and infographics. JL contributed to the conception, design, and contribution to data interpretation. JS performed experimental work (DC transfection) and contributed to data interpretation. AS performed experimental work (optimization of adherent PBMCs isolation protocol) and revision of the manuscript. AM performed experimental work (preparation of DNA constructs). SS contributed to the conception, design, revision, and final approval of the manuscript. All authors read and approved the final version of this manuscript.

### Conflict of Interest Statement

The authors declare that the research was conducted in the absence of any commercial or financial relationships that could be construed as a potential conflict of interest.

## References

[1] Peters PJ Borst J Oorschot V Fukuda M Krähenbühl O Tschopp J. Cytotoxic T lymphocyte granules are secretory lysosomes, containing both perforin and granzymes. J Exp Med. (1991) 173:1099–109. 10.1084/jem.173.5.1099 2022921 PMC2118839

[2] Barry M Bleackley RC. Cytotoxic T lymphocytes: all roads lead to death. Nat Rev Immunol. (2002) 2:401–9. 10.1038/nri819 12093006

[3] Halle S Halle O Förster R. Mechanisms and dynamics of T cell-mediated cytotoxicity *in vivo*. Trends Immunol. (2017) 38:432–43. 10.1016/j.it.2017.04.002 28499492

[4] Sommermeyer D Hudecek M Kosasih PL Gogishvili T David G Turtle CJ. Chimeric antigen receptor-modified T cells derived from defined CD8+ and CD4+ subsets confer superior antitumor reactivity *in vivo*. Leukemia. (2016) 30:492–500. 10.1038/leu.2015.247.Chimeric 26369987 PMC4746098

[5] de Wolf C van de Bovenkamp M Hoefnagel M. Regulatory perspective on *in vitro* potency assays for human T cells used in anti-tumor immunotherapy. Cytotherapy. (2018) 20:601–22. 10.1016/j.jcyt.2018.01.011 29598903

[6] Perica K Varela JC Oelke M Schneck J. Adoptive T cell immunotherapy for cancer. Rambam Maimonides Med J. (2015) 6:e0004. 10.5041/RMMJ.10179 25717386 PMC4327320

[7] Gattinoni L Powell DJ Rosenberg SA Restifo NP. Adoptive immunotherapy for cancer: building on success. Nat Rev Immunol. (2006) 6:383–93. 10.1038/nri1842 16622476 PMC1473162

[8] June CH. Adoptive T cell therapy for cancer in the clinic. J Clin Invest. (2007) 117:1466–76. 10.1172/JCI32446 17549249 PMC1878537

[9] Ramírez N Beloki L Ciaúrriz M Rodríguez-Calvillo M Escors D Mansilla C. Impact of T cell selection methods in the success of clinical adoptive immunotherapy. Cell Mol Life Sci. (2013) 71:1211–24. 10.1007/s00018-013-1463-5 24077876 PMC11113470

[10] Perret R Ronchese F. Memory T cells in cancer immunotherapy : which CD8 + T-cell population provides the best protection against tumours. (2008) 72:187–94. 10.1111/j.1399-0039.2008.01088.x 18627571

[11] Gutierrez C Schiff R. HER2: biology, detection, and clinical implications. Arch Pathol Lab Med. (2011) 135:55–62. 10.1043/2010-0454-RAR.1 21204711 PMC3242418

[12] Wen YY Hu XS. Anti-tumor activity of dendritic cell-cytokine induced killer cells (DC-CIks) sensitized to HER2 against HER-positive breast cancer cells. Genet Mol Res. (2016) 15:1–8. 10.4238/gmr.15027853 27173208

[13] Rongcun Y Salazar-Onfray F Charo J Malmberg KJ Evrin K Maes H. Identification of new HER2/neu-derived peptide epitopes that can elicit specific CTL against autologous and allogeneic carcinomas and melanomas. J Immunol. (1999) 163:1037–44. 10395702

[14] Goebel SU Iwamoto M Raffeld M Gibril F Hou W Serrano J. HER-2/neu expression and gene amplification in gastrinomas: correlations with tumor biology, growth, and aggressiveness. Cancer Res. (2002) 62:3702–10. 12097278

[15] Eroglu Z Tagawa T Somlo G. Human epidermal growth factor receptor family-targeted therapies in the treatment of HER2-overexpressing breast cancer. Oncologist. (2014) 19:135–50. 10.1634/theoncologist.2013-0283 24436312 PMC3926785

[16] Bernhard H Neudorfer J Gebhard K Conrad H Hermann C Nährig J. Adoptive transfer of autologous, HER2-specific, cytotoxic T lymphocytes for the treatment of HER2-overexpressing breast cancer. Cancer Immunol Immunother. (2008) 57:271–80. 10.1007/s00262-007-0355-7 17646988 PMC11030865

[17] English DP Roque DM Santin AD. HER2 expression beyond breast cancer: therapeutic implications for gynecologic malignancies. Mol Diagn Ther. (2013) 17:85–99. 10.1007/s40291-013-0024-9 23529353 PMC3660991

[18] Disis ML Dang Y Coveler AL Marzbani E Kou ZC Childs JS. HER-2/neu vaccine-primed autologous T-cell infusions for the treatment of advanced stage HER-2/neu expressing cancers. Cancer Immunol Immunother. (2014) 63:101–9. 10.1007/s00262-013-1489-4. 24162107 PMC3945106

[19] Maksyutov AZ Lopatnikova YA Kurilin VV Shevchenko YA Khantakova YN Gavrilova EV Efficiency studies of induced cytotoxic immune response of mononuclear cells by means of dendritic cells transfected by polyepitopic HER2/ERBB2 constructs. Meditsinskaya Immunol. (2014) 16:417–24. 10.15789/1563-0625-2014-5-417-424

[20] Subbiah IM Gonzalez-Angulo AM. Advances and future directions in the targeting of HER2-positive breast cancer: implications for the future. Curr Treat Options Oncol. (2014) 15:41–54. 10.1007/s11864-013-0262-4 24323591 PMC3933950

[21] Grela-Wojewoda A Niemiec J Sas-Korczynska B Cedrych I Domagała-Haduch M Adamczyk A. Prognostic role of nodal status and clinically asymptomatic valvular insufficiency in patients with HER2-positive breast cancer treated with chemotherapy, radiotherapy and trastuzumab in an adjuvant setting. Anticancer Res. (2015) 35:4063–72. 26124356

[22] Ahmed N Brawley VS Hegde M Robertson C Ghazi A Gerken C. Human epidermal growth factor receptor 2 (HER2) - Specific chimeric antigen receptor - Modified T cells for the immunotherapy of HER2-positive sarcoma. J Clin Oncol. (2015) 33:1688–96. 10.1200/JCO.2014.58.0225 25800760 PMC4429176

[23] Kuznetsova M Lopatnikova J Khantakova J Maksyutov R Maksyutov A Sennikov S. Generation of populations of antigen-specific cytotoxic T cells using DCs transfected with DNA construct encoding HER2/neu tumor antigen epitopes. BMC Immunol. (2017) 18:31. 10.1186/s12865-017-0219-7 28633645 PMC5479015

[24] Jiao P Zhang J Dong Y Wei D Ren Y. Construction and characterization of the recombinant immunotoxin RTA-4D5-KDEL targeting HER2/neu-positive cancer cells and locating the endoplasmic reticulum. Appl Microbiol Biotechnol. (2018) 102:9585–94. 10.1007/s00253-018-9291-z 30141083

[25] Takata H Takiguchi M. Three memory subsets of human CD8+ T cells differently expressing three cytolytic effector molecules. J Immunol. (2006) 177:4330–40. 10.4049/jimmunol.177.7.4330 16982867

[26] Mahnke YD Brodie TM Sallusto F Roederer M Lugli E. The who's who of T-cell differentiation: human memory T-cell subsets. Eur J Immunol. (2013) 43:2797–809. 10.1002/eji.201343751 24258910

[27] Kudryuavtsev IV Memory T cells: major populations and stages of differentiation. Russian J Immunol. (2014) 8:947–64.

[28] Samji T Khanna KM. Understanding memory CD8+ T cells. Immunol Lett. (2017) 185:32–9. 10.1016/j.imlet.2017.02.012 28274794 PMC5508124

[29] Chen KY Liu J Ren EC. Structural and functional distinctiveness of HLA-A2 allelic variants. Immunol Res. (2012) 53:182–90. 10.1007/s12026-012-8295-5 22434516

[30] Middleton D Williams F Meenagh A Daar AS Gorodezky C Hammond M. Analysis of the distribution of HLA-A alleles in populations from five continents. Hum Immunol. (2000) 61:1048–52. 10.1016/S0198-8859(00)00178-6 11082518

[31] Woll MM Fisher CM Ryan GB Gurney JM Storrer CE Ioannides CG. Direct measurement of peptide-specific CD8+ T cells using HLA-A2:Ig dimer for monitoring the *in vivo* immune response to a HER2/neu vaccine in breast and prostate cancer patients. J Clin Immunol. (2004) 24:449–61. 10.1023/B:JOCI.0000029117.10791.98 15163902

[32] Mitchell DA Nair SK Nucleic acid therapeutics RNA-transfected dendritic cells in cancer immunotherapy. J Clin Invest. (2000) 106:1065–9. 10.1172/JCI11405 11067858 PMC301423

[33] Banchereau J Palucka AK. Dendritic cells as therapeutic vaccines against cancer. Nat Rev Immunol. (2005) 5:296–306. 10.1038/nri1592 15803149

[34] Kulikova EV Kurilin VV Shevchenko JA Obleukhova IA Khrapov EA Boyarskikh UA. Dendritic cells transfected with a DNA construct encoding tumour-associated antigen epitopes induce a cytotoxic immune response against autologous tumour cells in a culture of mononuclear cells from colorectal cancer patients. Scand J Immunol. (2015) 82:110–7. 10.1111/sji.12311 25966778

[35] Tüting T DeLeo AB Lotze MT Storkus WJ. Genetically modified bone marrow-derived dendritic cells expressing tumor-associated viral or “self” antigens induce antitumor immunity *in vivo*. Eur J Immunol. (1997) 27:2702–7. 10.1002/eji.1830271033 9368629

[36] Brunner S Fürtbauer E Sauer T Kursa M Wagner E Overcoming the nuclear barrier: cell cycle independent nonviral gene transfer with linear polyethylenimine or electroporation. Mol Ther. (2002) 5:80–6. 10.1006/mthe.2001.0509 11786049

[37] Bradburne C Robertson K Thach D. Assessment of methods and analysis of outcomes for comprehensive optimization of nucleofection. Genet Vaccines Ther. (2009) 7:6. 10.1186/1479-0556-7-6 19432988 PMC2683797

[38] Friedman KM Prieto PA Devillier LE Gross CA Yang JC Wunderlich JR. Tumor-specific CD4+ melanoma tumor-infiltrating lymphocytes. J Immunother. (2012) 35:400–8. 10.1097/CJI.0b013e31825898c5 22576345 PMC7412749

[39] Matsuzaki J Tsuji T Luescher IF Shiku H Mineno J Okamoto S. Direct tumor recognition by a human CD4+T-cell subset potently mediates tumor growth inhibition and orchestrates anti-tumor immune responses. Sci Rep. (2015) 5:14896. 10.1038/srep14896 26447332 PMC4597193

[40] Moeller M Kershaw MH Cameron R Westwood JA Trapani JA Smyth MJ. Sustained antigen-specific antitumor recall response mediated by gene-modified CD4+T helper-1 and CD8+T cells. Cancer Res. (2007) 67:11428–37. 10.1158/0008-5472.CAN-07-1141 18056471

[41] Turtle CJ Riddell SR Maloney DG Turtle CJ Hanafi L Berger C CD19 CAR – T cells of defined CD4 + : CD8 + composition in adult B cell ALL patients find the latest version : CD19 CAR – T cells of defined CD4 + : CD8 + composition in adult B cell ALL patients. J Clin Invest. (2016) 126:2123–38. 10.1172/JCI85309.modified 27111235 PMC4887159

[42] Fritsch RD Shen X Sims GP Hathcock KS Hodes RJ Lipsky PE. Stepwise differentiation of CD4 memory T cells defined by expression of CCR7 and CD27. J Immunol. (2005) 175:6489–97. 10.4049/jimmunol.175.10.6489 16272303

[43] Okada R Kondo T Matsuki F Takata H Takiguchi M. Phenotypic classification of human CD4+ T cell subsets and their differentiation. Int Immunol. (2008) 20:1189–99. 10.1093/intimm/dxn075 18635582

[44] Picker LJ Reed-Inderbitzin EF Hagen SI Edgar JB Hansen SG Legasse A. IL-15 induces CD4+ effector memory T cell production and tissue emigration in nonhuman primates. J Clin Invest. (2006) 116:1514–24. 10.1172/JCI27564 16691294 PMC1459071

[45] Lugli E Goldman CK Perera LP Smedley J Pung R Yovandich JL. Transient and persistent effects of IL-15 on lymphocyte homeostasis in nonhuman primates. Blood. (2010) 116:3238–48. 10.1182/blood-2010-03-275438 20631381 PMC2995354

[46] Sallusto F Geginat J Lanzavecchia A. Central memory and effector memory T cell subsets: function, generation, and maintenance. Annu Rev Immunol. (2004) 22:745–63. 10.1146/annurev.immunol.22.012703.104702 15032595

[47] Li A Zhang Q Jiang J Yuan G Feng Y Hao J. Co-transplantation of bone marrow stromal cells transduced with IL-7 gene enhances immune reconstitution after allogeneic bone marrow transplantation in mice. Gene Ther. (2006) 13:1178–87. 10.1038/sj.gt.3302741 16598299

[48] Gattinoni L Zhong X Palmer DC Ji Y Hinrichs CS Yu Z. Wnt signaling arrests effector T cell differentiation and generates CD8+ memory stem cells. Nat Med. (2009) 15:808–13. 10.1038/nm.1982 19525962 PMC2707501

[49] Gattinoni L Lugli E Pos Z Paulos CM Quigley MF Almeida JR. A human memory T-cell subset with stem cell-like properties. Nat Med. (2011) 17:1290–7. 10.1038/nm.2446.A 21926977 PMC3192229

[50] Flynn JK Gorry PR. Stem memory T cells (TSCM)—their role in cancer and HIV immunotherapies. Clin Transl Immunol. (2014) 3:e20. 10.1038/cti.2014.16 25505968 PMC4232066

